# Metastatic Immune-Related Genes for Affecting Prognosis and Immune Response in Renal Clear Cell Carcinoma

**DOI:** 10.3389/fmolb.2021.794326

**Published:** 2022-01-28

**Authors:** Si Sun, Weipu Mao, Lilin Wan, Kehao Pan, Liting Deng, Lei Zhang, Guangyuan Zhang, Ming Chen

**Affiliations:** ^1^ Department of Urology, Zhongda Hospital, Southeast University, Nanjing, China; ^2^ Medical School, Southeast University, Nanjing, China; ^3^ Surgical Research Center, Institute of Urology, Southeast University Medical School, Nanjing, China; ^4^ Department of Urology, Nanjing Lishui District People’s Hospital, Zhongda Hospital Lishui Branch, Southeast University, Nanjing, China

**Keywords:** renal clear cell carcinoma, metastatic immune-related genes, prognosis, immunotherapy, biomarkers

## Abstract

**Background:** In renal clear cell carcinoma, a common cancer of the urinary system, 25–30% patients are metastatic at initial diagnosis and 20–30% patients have a tendency of recurrence and metastasis after local surgery. With the rapid development of tumor immunology, immune agents have brought new directions to tumor therapy. However, no relevant studies have explored the role of immune-related genes in kidney cancer metastasis.

**Methods:** Co-expressed metastatic immune-related differentially expressed genes (mIR-DEGs) were screened by GSE12606, GSE47352, and immunorelated genes. Then, differential expression analysis, prognostic analysis, and univariate and multivariate Cox regression analysis in KIRC were performed to determine independent prognostic factors associated, and the risk prognostic model was established. The correlation of hub mIR-DEGs with clinicopathological factors, immune invasion, and immune checkpoints was analyzed, and the expression of hub mIR-DEGs and their effect on tumor were re-evaluated by OCLR scores in KIRC.

**Results:** By comprehensive bioassay, we found that FGF17, PRKCG, SSTR1, and SCTR were mIR-DEGs with independent prognostic values, which were significantly associated with clinicopathological factors and immune checkpoint–related genes. The risk prognostics model built on this basis had good predictive potential. In addition, targeted small molecule drugs, including calmidazolium and sulfasalazine, were predicted for mIR-DEGs. Further experimental results were consistent with the bioinformatics analysis.

**Conclusion:** This study preliminarily confirmed that FGF17, PRKCG, SSTR1, and SCTR were targeted genes affecting renal cancer metastasis and related immune responses and can be used as potential therapeutic targets and prognostic biomarkers for renal cancer. Preliminary validation found that PRKCG and SSTR1 were consistent with predictions.

## Introduction

Renal cell carcinoma (RCC) is the most common renal malignancy originating from tubular epithelium (Siegel et al., 2018). Kidney renal clear cell carcinoma (KIRC) accounts for approximately 80% of all clinical cases of renal cell carcinoma in adults and is the most common histological subtype (Ricketts et al., 2018). In the 2021 Global Cancer Statistics, RCC accounted for approximately 4% of all newly diagnosed cancers, ranking sixth among cancers in men and ninth among cancers in women (Siegel et al., 2021). 25%–30% of patients are metastatic at initial diagnosis (Ljungberg et al., 2011), and 20–30% of patients tend to have recurrence and metastasis after local surgery (Athar and Gentile, 2008; Mao et al., 2021a). Due to resistance to radiation and chemotherapy (Braun et al., 2021), surgical resection is still the best treatment for RCC (Escudier et al., 2019).

In recent years, the treatment of RCC has made rapid progress. Much evidence has confirmed that RCC is highly immunogenic (Şenbabaoğlu et al., 2016) and is highly responsive to immunotherapy (Escudier, 2012). Among the most advanced therapies, immunotherapy can effectively and safely treat tumors (Xie et al., 2019; Frega et al., 2020). Its characteristic is to stimulate specific immune response and inhibit and kill tumor cells, thereby reducing tumor metastasis and recurrence. As an indispensable part of immunotherapy, the tumor immune microenvironment (TIME) has attracted more and more attention. The tumor is always in a complex tissue microenvironment, and the changes of immune microenvironment may affect the occurrence, development, and metastasis of tumor in different ways. The analysis of the immune microenvironment will help improve the response of immunotherapy. Some researchers have found that the TIME can be used as an important prognostic indicator, which could also enhance the potential of precision therapy (Taube et al., 2018; Vuong et al., 2019). Although the advent of immunotherapy and targeted therapy has diversified the treatment of RCC, some patients with RCC develop symptoms only when their cancer cells have metastasized to a distant point in their body, and the five-year survival rate of these patients is usually less than 20% (Dunnick, 2016). The prognosis for patients with renal cell carcinoma remains dismal. Therefore, it is urgent to search for targeted biomarkers related to metastasis and immunity in RCC.

In this study, the comprehensive bioinformatics analysis of GSE12606 (Stickel et al., 2009), GSE47352 (Gao et al., 2017), and immune-related genes was performed, and independent prognostic factors were identified by differential expression analysis, survival analysis, and univariate and multivariate Cox regression analysis, which contributed to renal cancer metastasis. The good prognostic risk model was constructed based on metastatic immune-related independent prognostic genes. In addition, we found that hub target genes were closely associated with the tumor immune microenvironment and immune checkpoint genes. Based on the target gene, we successfully predicted the potential therapeutic drugs to prevent renal cancer progression and assist immunotherapy. In conclusion, this study provided insights into immune-related molecular mechanisms underlying the progression of renal cancer from primary to metastatic stage and identified biomarkers that might have prognostic value.

## Materials and Methods

### Screening of IR-DEGs in Primary and Metastatic KIRC

To acquire metastatic immune-related differentially expressed genes (mIR-DEGs) in primary and metastatic kidney renal clear cell carcinoma (KIRC), we used the GEO database (https://www.ncbi.nlm.nih.gov). The GSE12606 and GSE47352 datasets were selected for subsequent analysis (Supplementary Table S1). The cut-off conditions were set to *p*-value < 0.05, and the absolute value of log-fold change (|log_2_FC|) ≥ 1, which had been adjusted for multiple testing *via* the Benjamini–Hochberg procedure, was statistically significant for the DEGs. We use ImageGP to create volcano maps and Venn maps online.

### Functional Enrichment Analysis of mIR-DEGs

Enrichment analysis of mIR-DEGs was performed by Gene Ontology (GO) and Kyoto Encyclopedia of Genes and Genomes (KEGG) pathway analysis in the “ClusterProfiler” package.

### Identification of Independent Prognostic mIR-DEGs

Univariate and multivariate Cox regression analyses were performed on mIR-DEGs, and the forest maps were established by the “Forestplot” R package. The univariate Cox regression analysis result was included in the multivariate regression analysis when *p* threshold value < 0.05, and the independent prognostic genes were finally identified with *p* < 0.005. RNA sequencing data of 539 renal clear cell carcinoma samples and 72 paracancerous samples, obtained from The Cancer Genome Atlas (TCGA) database (https://cancergenome.nih.gov/), were used to evaluate mIR-DEGs’ expression and prognosis by Gene Expression Profiling Interactive Analysis (GEPIA) (http://gepia2.cancer-pku.cn/index) and “survival” package. The basic information of TCGA-KIRC patients is listed in Supplementary Table S2.

### Construction and Validation of the Hub mIR-DEGs’ Prognostic Model

Hub mIR-DEGs were selected based on univariate and multivariate Cox regression analysis, differential expression analysis, and prognostic analysis of mIR-DEGs. The lasso Cox regression was used to construct the risk prognosis model of hub mIR-DEGs based on the “GLMnet” R package. Risk coefficients were calculated by centralized standardized analysis in TCGA: Risk Score = ∑7iXi × Yi (X: coefficients, Y: gene expression level). Then, t-distributed stochastic neighbor embedding (t-SNE) and principal-component analysis (PCA) were used to explore the distribution characteristics of different groups by R packages. Finally, the effectiveness of prognostic indicators was evaluated by the area under the curve (AUC) of “time receiver-operating characteristic (ROC) curve.” Furthermore, Spearman correlation analysis was used to explore the relationship between the model score and the immune score by QUANTISEQ, and the R software package Pheatmap was used to verify the relationship.

### Construction of Clinicopathological Correlation Analysis and the Nomogram

Based on the “survival” package in R software, combined with the clinicopathological characteristics of patients (TNM stage, pathological stage, histologic stage, and laterality), the correlation between FGF17, PRKCG, SSTR1, and SCTR in the prognostic model and clinicopathological characteristics was analyzed. Through the R package “rms,” the nomogram and calibration curve were obtained. Risk scores associated with prognostic models were used as prognostic factors to evaluate one-, three-, and five-year OS.

### Assessment of the Immune Microenvironment About Hub mIR-DEGs in KIRC

The correlation between FGF17, PRKCG, SSTR1, and SCTR expressions and copy number and various immune cells in KIRC were searched and analyzed through the gene module by TIMER (https://cistrome.shinyapps.io/timer/), including B cells, CD4+ T cells, CD8+ T cells, macrophages, neutrophils, and dendritic cells.

In this study, kidney cancer immune cells were investigated by the QUANTISEQ1-2 algorithm, which quantifies tumor immune status based on human RNA-seq data, and the proportion of different immune cells and other uncharacterized cells present in the sample by a deconvolution algorithm, including B cells, macrophages, M2 macrophages, monocytes, neutrophils, NK cells, CD4+ T cells, CD8+ T cells, Tregs, myeloid cells, and uncharacterized cells (Finotello et al., 2019; Plattner et al., 2020).

### Relationship Between Immune Checkpoint–Related Genes and Expression of Hub mIR-DEGs in KIRC

The relationship between SIGLEC15, TIGIT, CD274, HAVCR2, PDCD1, CTLA4, LAG3, and PDCD1LG2 and hub mIR-DEGs’ expression was analyzed using the “ggplot2” R package. Subsequently, the Tumor Immune Dysfunction and Exclusion (TIDE) algorithm was used to evaluate the potential ICB response of different hub mIR-DEGs’ expression levels to immune checkpoint inhibitors in KIRC.

### OCLR Scores of Hub mIR-DEGs in KIRC

Tumor-associated RNA-seq data were obtained from TCGA-KIRC, mRNAsi was calculated by the OCLR algorithm, and the dryness index was obtained.

### Prediction of Small Molecule Drugs for Hub mIR-DEGs

The hub mIR-DEGs were used for drug prediction in Connectivity Map (www.broadinstitute.org), which was commonly used to explore potential drugs for the treatment of diseases. Therefore, Enrichment > 0.7, *p* < 0.02, and Percent non-nulld >75 were used for screening. The PubChem22 database (www.pubchem.ncbi.nlm.nih.gov) was used to retrieve the molecular structure of identified drugs.

### Cell Lines, Patient Samples, RNA Extraction, and Quantitative Real-Time Polymerase Chain Reaction (qRT-PCR)

The human kidney cell line, HK-2, and human KIRC cell lines, 786-O and caki-1, were originally purchased from the cell repository of Shanghai Institute of Life Sciences. The cells were cultured in 1640 medium (GIBCO), containing 10% FBS (GIBCO), penicillin (25 U/ml), and streptomycin (25 mg/ml), and at 5% CO_2_ environment.

In this study, 19 fresh samples, including tumor tissue and adjacent normal kidney tissue, were collected from patients who underwent laparoscopic radical nephrectomy for KIRC from 2019 to 2020 in the Department of Urology, Zhongda Hospital, and stored at 80°C. All patients were diagnosed with KIRC and did not receive any antitumor therapy preoperatively. Clinical characteristics of 19 KIRC patients are listed in Supplementary Table S3. The methodology of this study followed the criteria outlined in the Helsinki Declaration (revised in 2013), and ethical approval was obtained from the Ethics Committee and Institutional Review Board for Clinical Research of Zhongda Hospital (ZDKYSB077). All patients or their relatives who participated were informed and signed an informed consent form.

Total RNA was isolated with Total RNA Kit (OMEGAbiotec, Guangzhou, China) according to the manufacturer’s instructions. Complementary DNA was synthesized using the HiScript II Q RT SuperMix (R223-01) reagent kit (Vazyme Biotech Co., Ltd., Nanjing, China). The qRT-PCR was performed using the SYBR Green PCR Mix (Vazyme Biotech Co., Ltd., Nanjing, China). The specific primers set for mIR-DEGs and GAPDH are listed in Supplementary Table S4. Data were normalized to GAPDH expression levels using the 2^−ΔΔCt^ method.

### Tissue Microarray Construction and Immunohistochemistry

All specimens were fixed in 10% neutral formaldehyde solution and embedded in paraffin. Envision two-step dyeing and DAB color development were used. The primary antibody (FGF17, ab187982, Abcam; PRKCG, ab181558, Abcam; SSTR1, ab140945, Abcam) was used in this study.

### Statistical Analysis

The statistical analysis was carried out by R software (version 4.0.2). The Perl programming language (version 5.30.2) was used for data processing. Multivariate Cox regression analyses were used to evaluate prognostic significance. When *p* < 0.05 or log-rank *p* < 0.05, the difference was statistically significant.

## Results

### Identification of mIR-DEGs

**FIGURE 1:**
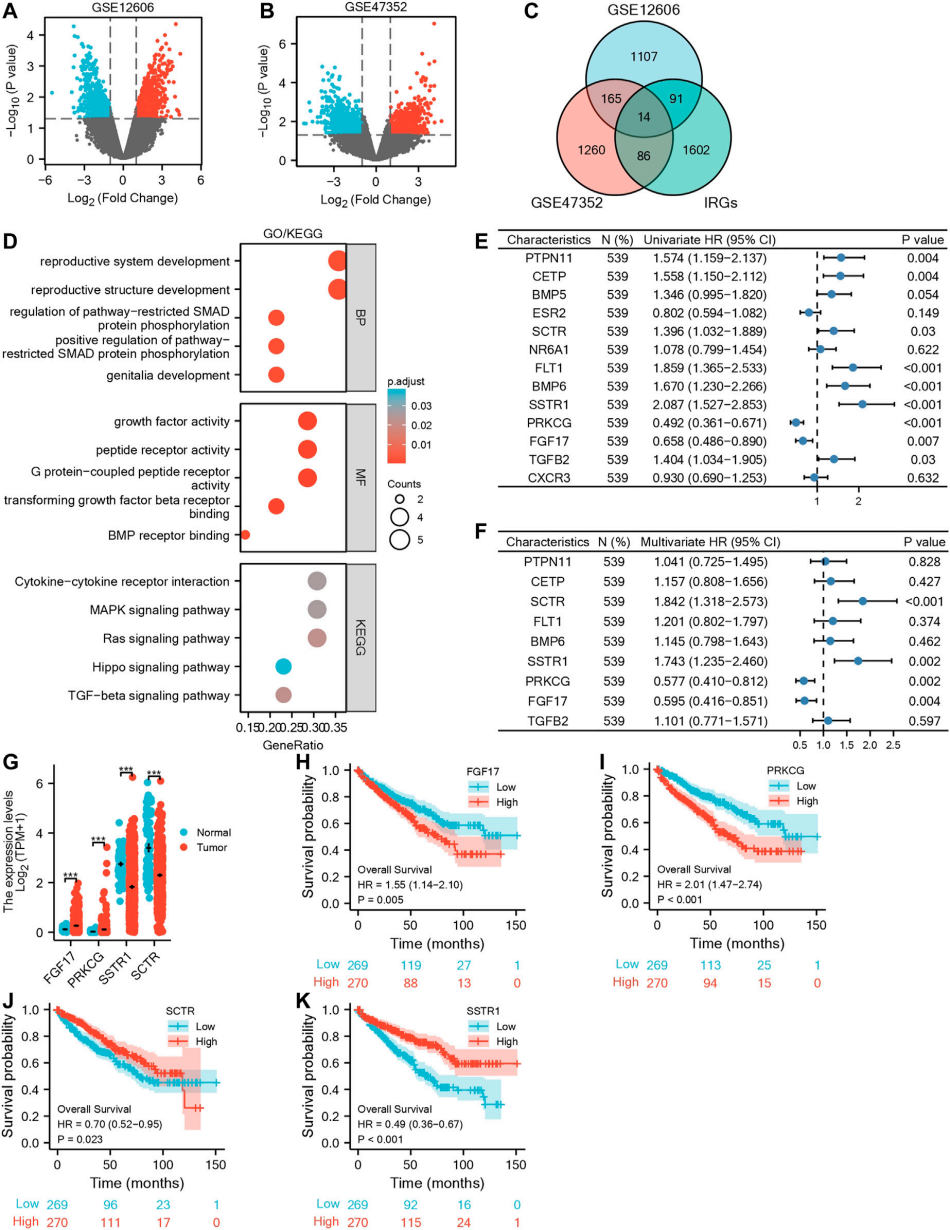
Screening for independent prognostic genes in KIRC. **(A,B)** Volcano maps of GSE12606 and GSE47352. **(C)** Venn diagram of GSE12606, GSE47352, and immune-related genes. **(D)** GO|KEGG enrichment analysis of IR-DEGs. **(E,F)** Forest plots of univariate and multivariate Cox regression analysis of IR-DEGs. **(G)** Based on the GEPIA database for differential expression of the four IR-DEGs. **(H–K)** Survival analysis of four IR-DEGs, including SCTR **(H)**, SSTR1 **(I)**, PRKCG **(J)**, and FGF17 **(K)**.

Sequencing data related to primary and metastatic renal carcinoma were obtained from the GEO database (Figures 1A,B). 1,377 metastatic DEGs (mDEGs) were screened in GSE12606, and 1,525 mDEGs were screened in GSE47352. Then, the mDEGs and 1,793 immune-related genes, which are from the ImmPort database, were analyzed by Venn diagram, and 14 co-expressed genes were obtained by the intersection of the three gene sets (Figure 1C). Then, through GO/KEGG pathway enrichment analysis (Supplementary Table S5), it was found that the functions of 14 mIR-DEGs were mainly concentrated in “reproductive structure development,” “reproductive system development,” “positive regulation of pathway-restricted SMAD protein phosphorylation,” “G protein-coupled peptide receptor activity,” “peptide receptor activity,” “growth factor activity,” “TGF-beta signaling pathway,” “MAPK signaling pathway,” and “Cytokine-cytokine receptor interaction” (Figure 1D). To further clarify the correlation between mIR-DEGs and prognosis, univariate and multivariate Cox regression analysis (Supplementary Table S6) showed that FGF17, PRKCG, SSTR1, and SCTR were independent prognostic factors for the progression of KIRC from primary to metastatic stage (Figures 1E,F). Consequently, after screening hub mIR-DEGs with stringent criteria, the results conform to the Bonferroni correction significant level and minimize the inflation of Type I errors from multiple testing issues.

### Differential Expression Analysis and Survival Analysis of Hub mIR-DEGs in KIRC

Using the TCGA-KIRC database, we verified the expression levels of four mIR-DEGs that were significant in univariate Cox regression analysis and found the expression levels of PRKCG and FGF17 were up-regulated and SCTR and SSTR1 were down-regulated in 539 tumors and 72 paracancerous samples (Figure 1G). Then, Kaplan–Meier model analysis showed that the above four mIR-DEGs were significantly associated with prognosis, and the high expressions of SCTR and SSTR1 and TGFB2 were associated with good prognosis (Figures 1J,K), while the high expressions of PRKCG and FGF17 were significantly associated with poor prognosis (Figures 1H,I). Combined with multivariate Cox regression analysis, FGF17, PRKCG, SSTR1, and SCTR were identified as the hub metastatic immune-related independent prognostic factors, which influenced the progression of primary to metastatic kidney cancer.

### Construction and Validation of the Hub mIR-DEGs’ Prognostic Risk Model

**FIGURE 2:**
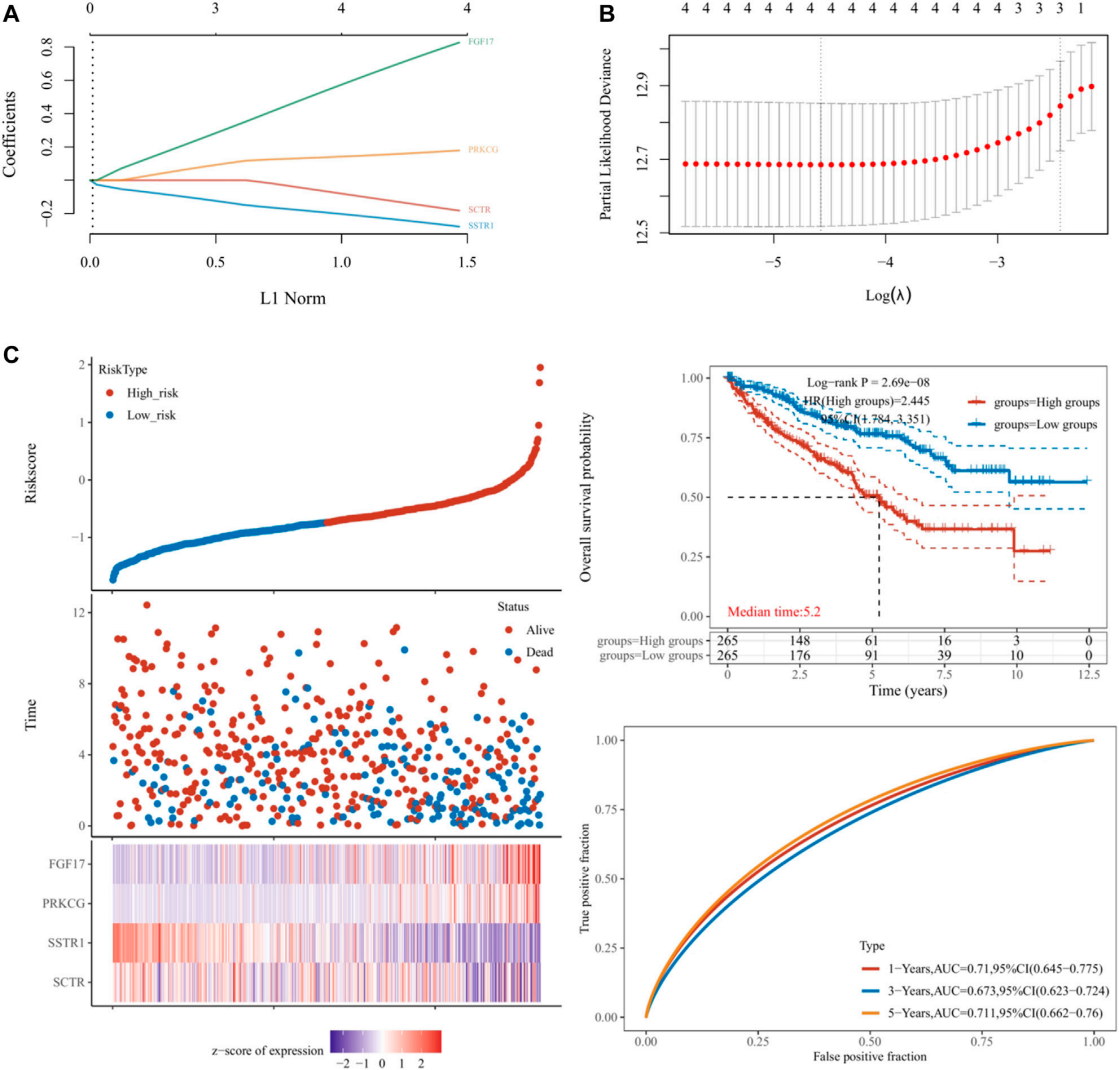
Establishment and validation of prognostic models in KIRC. **(A,B)** Lasso regression analysis results. **(C)** Risk score distribution, survival status, and four hub IR-DEGs in low- and high-risk groups. Kaplan–Meier survival curve of two groups. Time-dependent ROC curve analyses in TCGA set.

**FIGURE 3:**
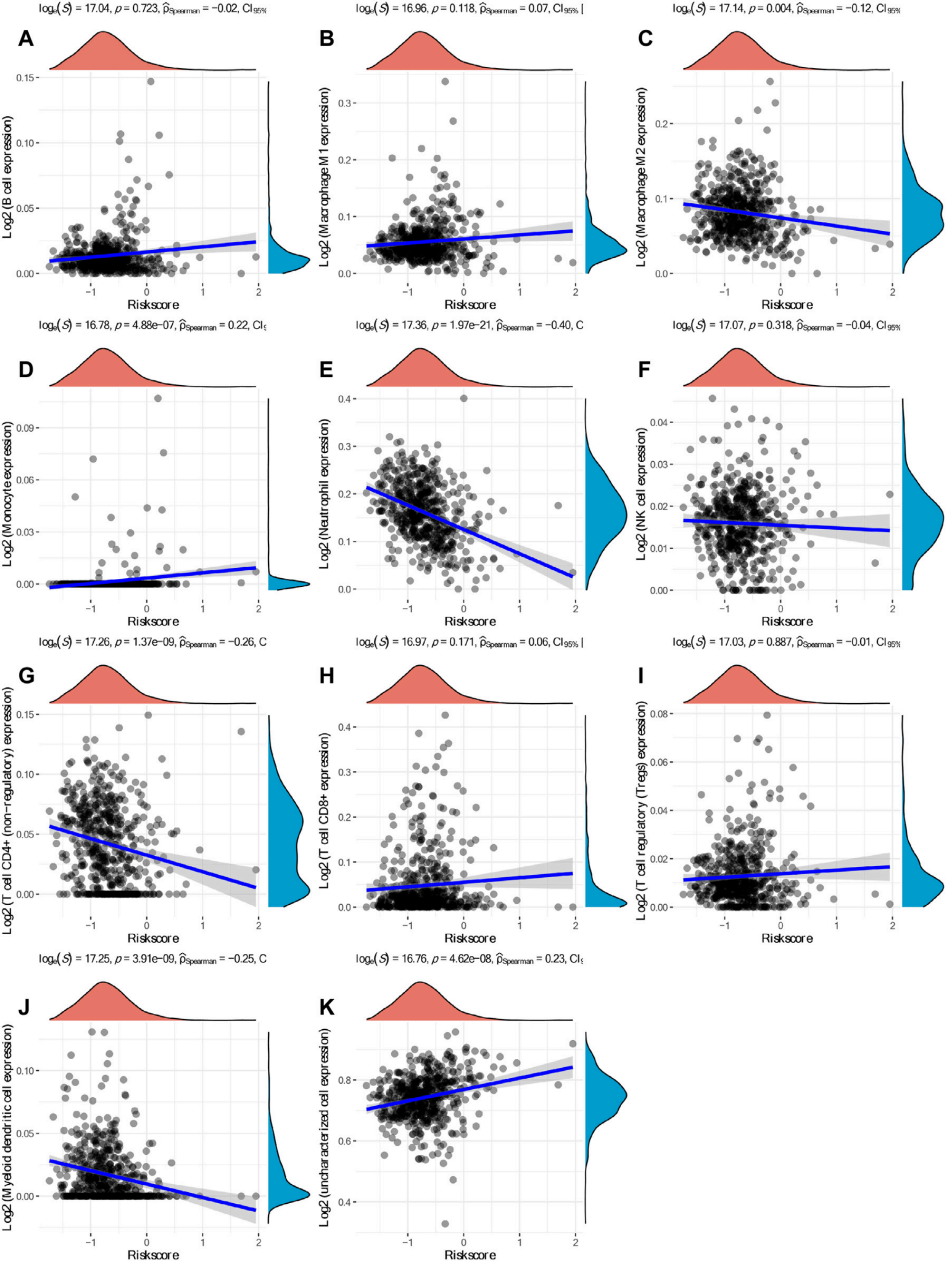
Spearman correlation analysis between the model score and the immune score. **(A)** B cells. **(B)** M1 macrophages. **(C)** M2 macrophages. **(D)** Monocytes. **(E)** Neutrophils. **(F)** NK cells. **(G)** CD4+ T cells. **(H)** CD8+ T cells. **(I)** Tregs. **(J)** Myeloid dendritic cells. **(K)** Uncharacterized cells.

Based on hub mIR-DEGs, lasso Cox regression was used to construct relevant risk prognosis models, lambda.min = 0.0103, Risk Score= (−0.1637) × SCTR + (−0.2632) × SSTR1 + (0.1711) × PRKCG + (0.7824) × FGF17 (Figures 2A,B). Patients were assigned into high-risk and low-risk groups according to the median risk score (50%). Survival status and hub mIR-DEGs’ heatmaps in different groups were displayed by t-SNE and PCA, indicating that FGF17 and PRKCG were highly expressed in the high-risk group, while SSTR1 and SCTR were lowly expressed in the high-risk group (Figure 2C). The prognostic model was the risk factor model due to HR = 2.445, and the median survival time of the high-risk group was significantly shorter than that of the low-risk group (Figure 2C). Finally, we evaluated the prognostic prediction efficiency of the model by the ROC curve. We found that the AUC was 0.71 (one-year OS), 0.673 (three-year OS), and 0.711 (five-year OS), respectively (Figure 2C). In addition, Spearman correlation analysis was used to explore the correlation between the hub mIR-DEGs risk prognosis model and the tumor immune microenvironment in KIRC (Figures 3A–K). The risk prognosis model was significantly negatively correlated with the infiltration of M2 macrophages (*r* = −0.12, *p* = 0.004), neutrophils (*r* = −0.40, *p* = 1.97e-21), CD4+ T cells (*r* = −0.26, *p* = 0.1.37e-09), and myeloid dendritic cells (*r* = −0.25, *p* = 3.91e-09) (Figures 3C,E,G,J) and significantly positively correlated with the infiltration of monocytes (*r* = 0.22, *p* = 4.88e-07) and uncharacterized cells (*r* = 0.23, *p* = 4.62e-08) (Figures 3D,K). These results indicated that the hub mIR-DEG–based risk prognosis model, including FGF17, PRKCG, SSTR1, and SCTR, had good predictive effect and was significantly correlated with the KIRC immune microenvironment.

### Relationship Between Hub mIR-DEGs and Clinicopathological Factors and the Construction Nomogram

**FIGURE 4:**
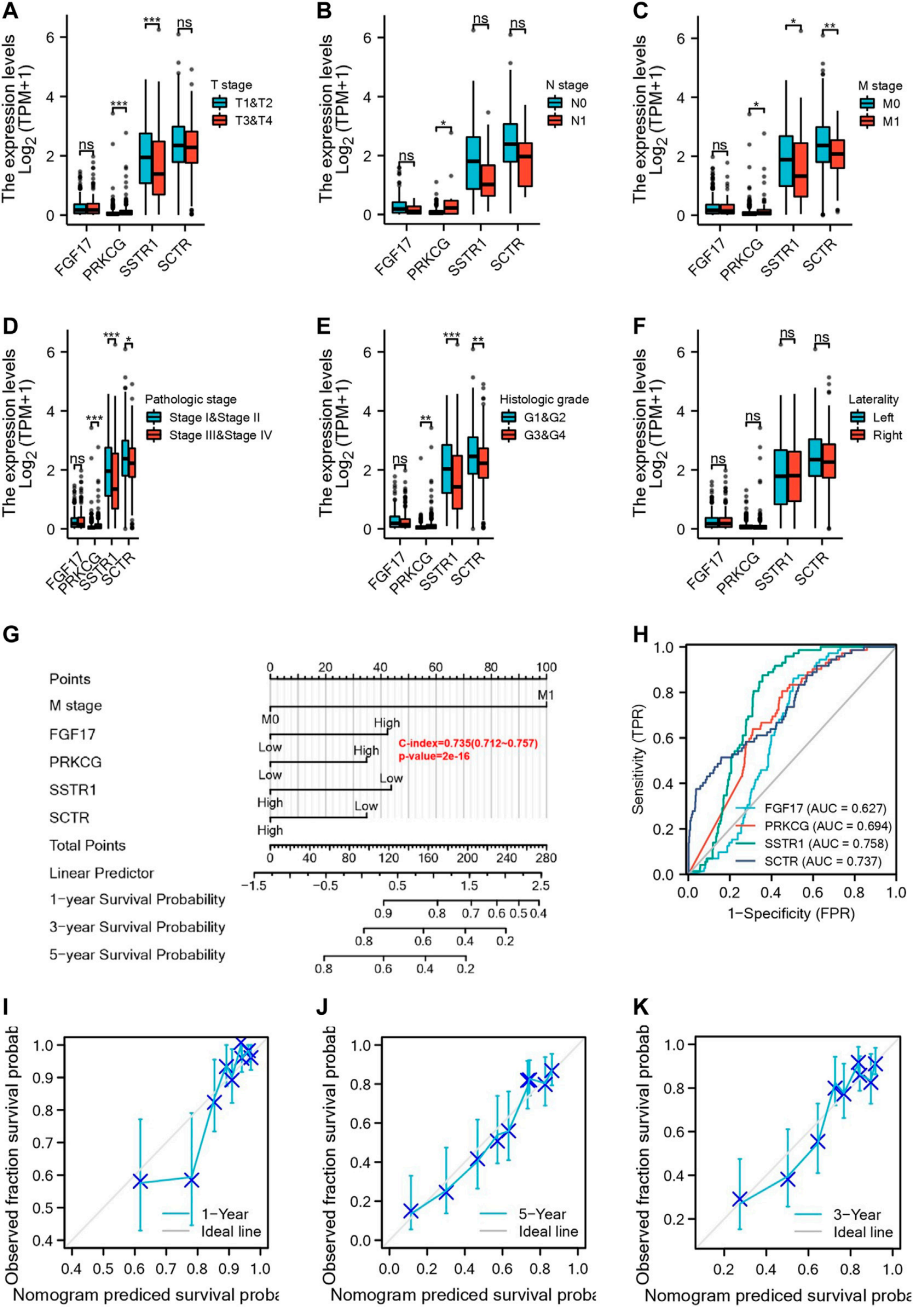
Four IR-DEGs correlate with multiple clinicopathological factors in KIRC. Relationships between IR-DEGs and clinicopathological factors in the entire TCGA cohort, including T stage **(A)**, N stage **(B)**, M stage **(C)**, histologic grade **(D)**, pathologic stage **(E)**, and laterality **(F)**. **(G)** Nomogram for predicting one‐, three‐, and five-year OS in the entire TCGA cohort. **(H–K)** Calibration curves of nomogram on consistency between predicted and observed one‐, three‐, and five-year survival in the entire TCGA cohort. The dashed line at 45° implies a perfect prediction, and the actual performances of our nomogram are shown by blue lines.

We analyzed the correlation of FGF17, PRKCG, SSTR1, and SCTR in the risk prognosis model with clinicopathological features. The results showed that the expression of PRKCG and SSTR1 was correlated with T stage (Figure 4A), PRKCG was correlated with N stage (Figures 4B,C), and the expression of PRKCG, SSTR1, and SCTR was associated with M stage, pathologic stage, and histologic stage (Figures 4D–F). One-, three-, and five-year OS was predicted by the nomogram, and the potential value of M stage for prognosis was determined in KIRC patients (Figure 4G). Subsequently, time-dependent ROC curve analysis showed that AUCFGF17 = 0.627, AUCPRKCG = 0.694, AUCSSTR1 = 0.758, and AUCSCTR = 0.737, indicating a good prognostic value of hub mIR-DEGs for KIRC patients (Figure 4H). In addition, we find that the calibration curve of the predicted probability was in good agreement with the one-, three-, and five-year OS on the nomogram, and the three-year OS was the best fit (Figures 4I–K).

### Assessment of the Immune Microenvironment About Hub mIR-DEGs in KIRC

**FIGURE 5:**
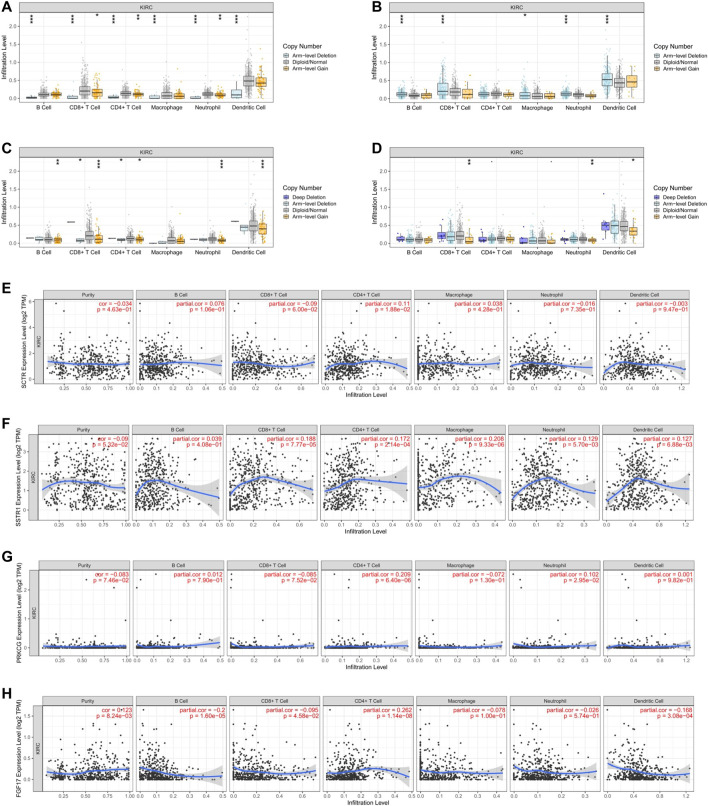
Relationship of immune cell infiltration with IR-DEG levels in KIRC. Infiltration level of various immune cells under different copy numbers of IR-DEG levels, including SCTR **(A)**, SSTR1 **(B)**, PRKCG **(C)**, and FGF17 **(D)**. Correlation of IR-DEG expression levels with B cell, CD8+ T cell, CD4+ T cell, macrophage, neutrophil, and dendritic cell infiltration levels, including SCTR **(E)**, SSTR1 **(F)**, PRKCG **(G)**, and FGF17 **(H)**.

In order to explore the potential relationship between the expression of FGF17, PRKCG, SSTR1, and SCTR in KIRC and the level of immune invasion, TIMER was used to conduct correlation analysis. First, we found positive correlations between SCTR and CD4+ T cells (*R* = 0.11, *p* = 1.88e-02). SSTR1 and CD8+ T cells (*R* = 0.188, *p* = 7.77e-05), CD4+ T cells (*R* = 0.172, *p* = 2.14e-04), macrophages (*R* = 0.208, *p* = 9.33e-06), neutrophils (*R* = 0.129, *p* = 5.70e-03), and DCs (*R* = 0.127, *p* = 6.88e-03) were positively correlated; PRKCG was positively correlated with CD4+ T cells (*R* = 0.209, *p* = 6.40e-06) and neutrophils (*R* = 0.102, *p* = 2.95e-02). FGF17 was positively correlated with CD4+ T cells (*R* = 0.262, *p* = 1.14e-08) but negatively correlated with B cells (*R* = −0.200, *p* = 1.60E-05) and DCs (*R* = −0.168, *p* = 3.08E-04) (Figures 5E–H). The copy numbers of SCTR, SSTR1, and PRKCG were significantly correlated with the infiltration levels of B cells, CD8+ T cells, CD4+ T cells, macrophages, neutrophils, and DCs (Figures 5A–C). However, FGF17 was only associated with CD8^+^ T cells, neutrophils, and DCs (Figure 5D).

### Relationship Between Immune Checkpoint–Related Genes and Expression of Hub mIR-DEGs

**FIGURE 6:**
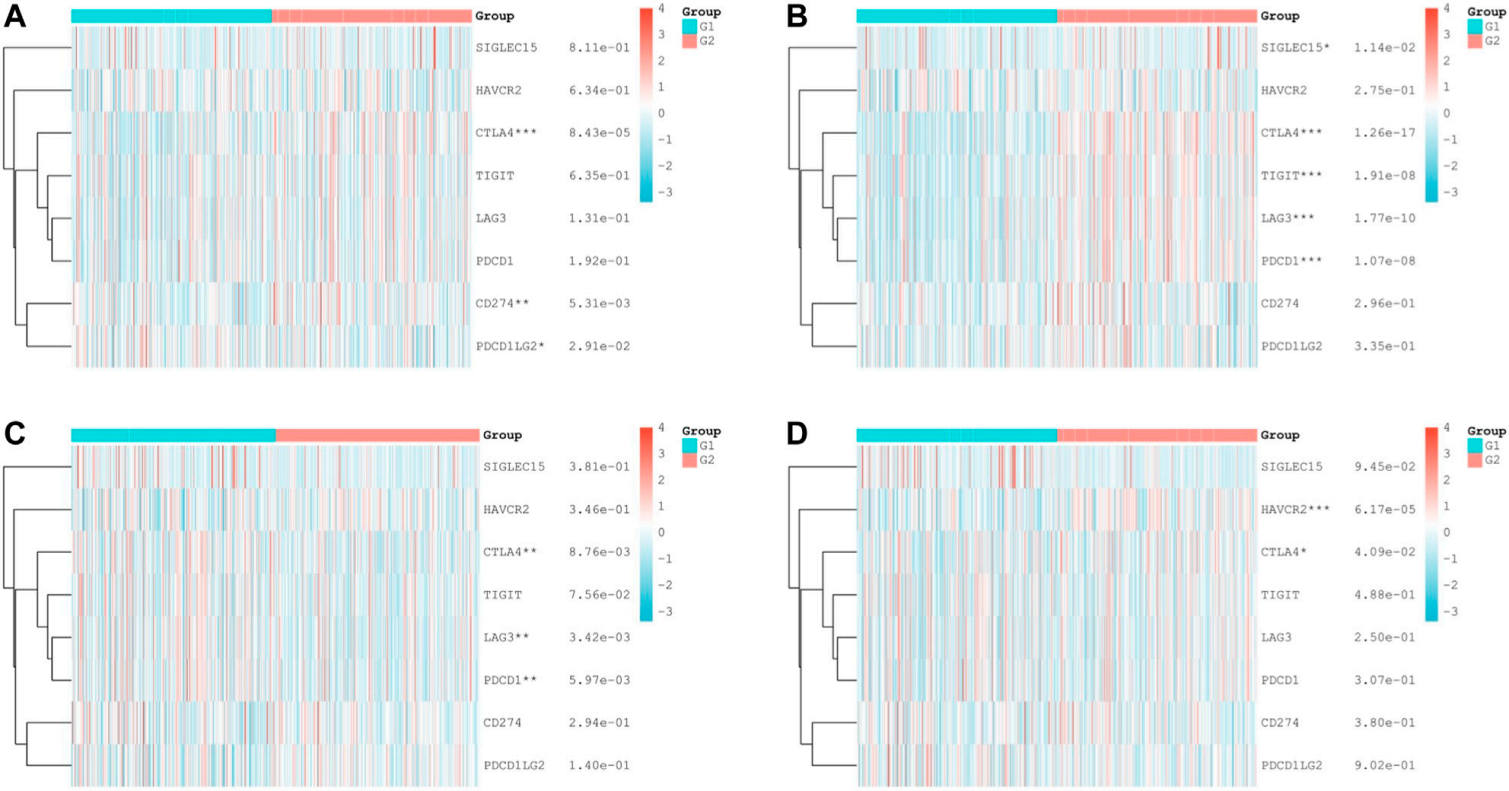
Differential expression of immune checkpoint—related genes in KIRC tissues. **(A–D)** Comparison of immune checkpoints in different expression levels of IR-DEGs and M stage in KIRC, including FGF17 **(A)**, PRKCG **(B)**, SSTR1 **(C)**, and SCTR **(D)**. G1 is IR-DEG upexpression in non-metastatic KIRC. G2 is IR-DEG downexpression in non-metastatic KIRC.

Based on the apparent correlation between hub mIR-DEGs, risk prediction models, and tumor immune microenvironment, we further explored the relationship between hub mIR-DEGs and immune checkpoints, providing potential directions for future immunotherapy (Figure 6). We found significant differences between FGF17 and CTLA4, CD274, and PDCD1LG2 (Figure 6A). PRKCG was significantly different from SIGLEC15, CTLA4, TIGIT, LAG3, and PDCD1 (Figure 6B). SSTR1 was significantly different from CTLA4, LAG3, and PDCD1 (Figure 6C). SCTR was significantly different from HAVCR2 and CTLA4 (Figure 6D). CTLA4 was strongly correlated with four hub mIR-DEGs. Our results suggested that CTLA4 might be a potential target for preventing KIRC progression and metastasis through immune checkpoint inhibitors in the risk prognosis model.

### Assessment of the OCLR Scores of Hub mIR-DEGs in KIRC

**FIGURE 7:**
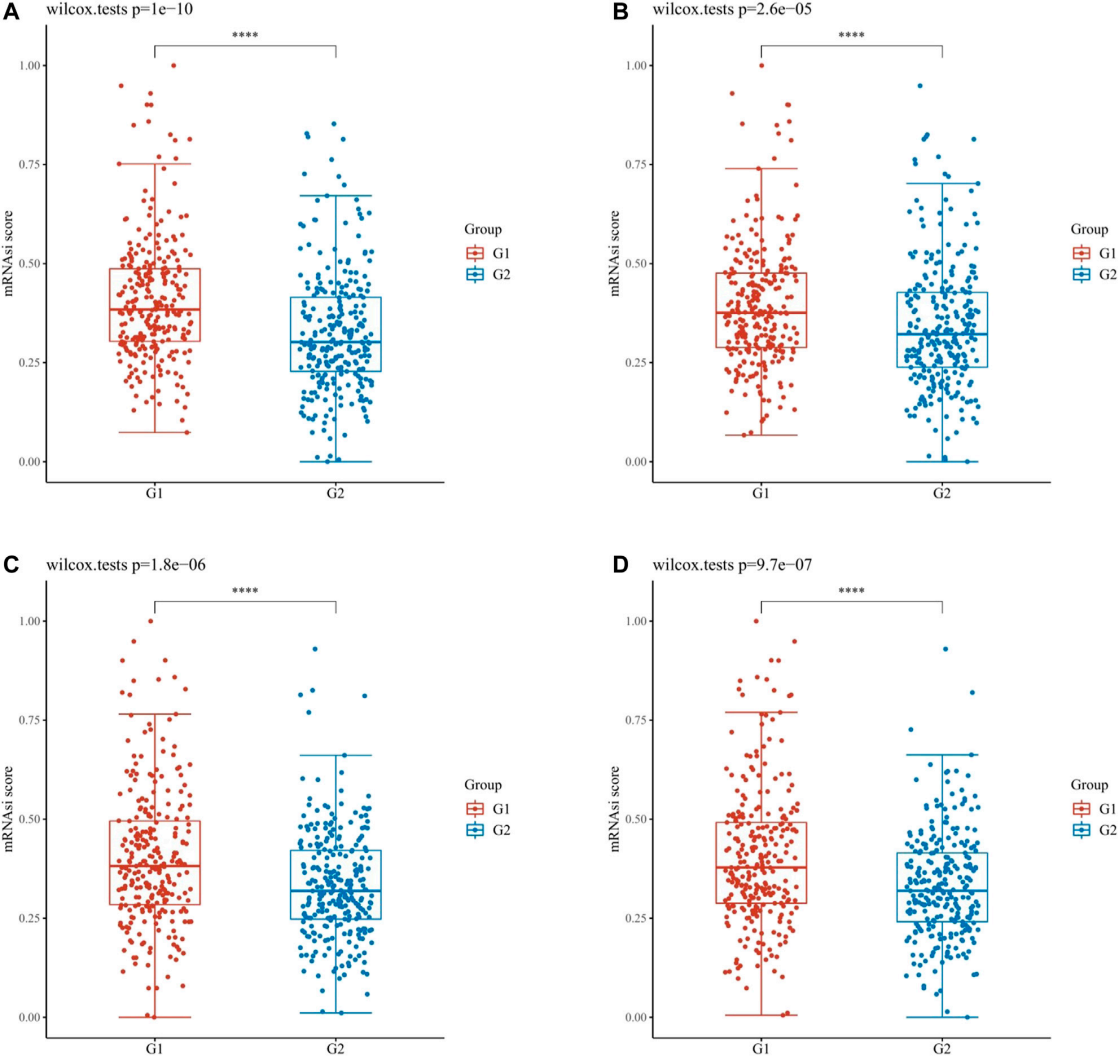
OCLR scores of hub IR-DEGs in KIRC. OCLR scores of hub IR-DEGs at different expression levels in KIRC, including FGF17 **(A)**, PRKCG **(B)**, SSTR1 **(C)**, and SCTR **(D)**. G1 is IR-DEG down-expression and G2 is up-expression in KIRC.

Through the dryness index, we discovered significant differences in dryness degree between hub mIR-DEGs in KIRC (Figure 7). These results suggested that FGF17, PRKCG, SSTR1, and SCTR might affect the degree of similarity between KIRC cells and stem cells, thus affecting tumor biological processes and degree of dedifferentiation.

### Prediction of Small Molecule Drugs for Hub mIR-DEGs

**FIGURE 8:**
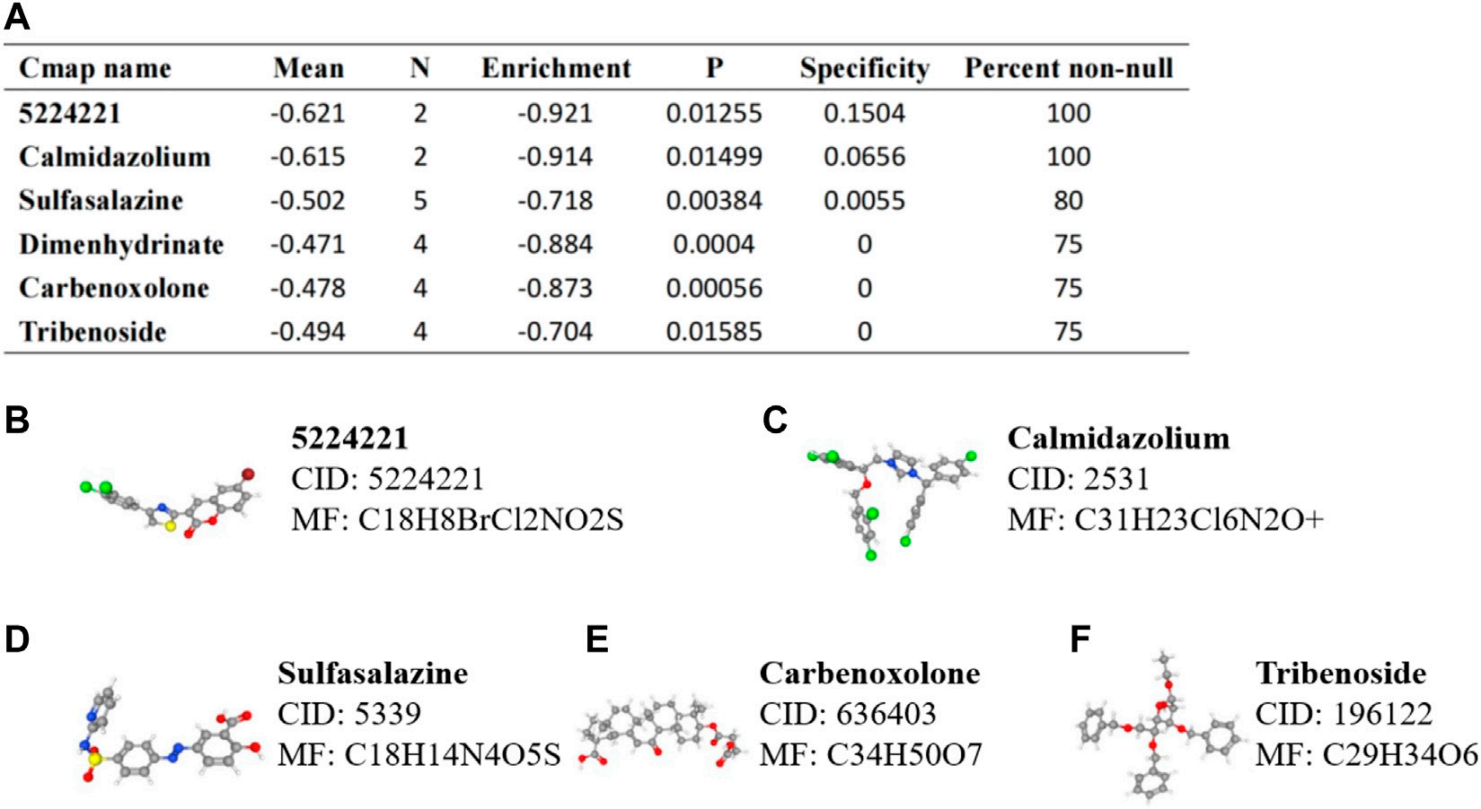
Prediction of small molecule drugs targeting IR-DEGs in KIRC. **(A)** mRNA probes were used to predict potential drugs for KIRC. **(B–F)** Prediction results of targeted drugs, including 5224221 **(B)**, calmidazolium **(C)**, sulfasalazine **(D)**, carbenoxolone **(E)**, and tribenoside **(F)**.

Based on the former analysis we performed, we can propose an assumption that FGF17, PRKCG, SSTR1, and SCTR had a potential role in the progression and metastasis of KIRC. Therefore, based on probes of FGF17 (221376_at), PRKCG (206270_at), SSTR1 (208482_at), and SCTR (210382_at), we predicted potential targeted drugs with immunotherapeutic effects and prevention of KIRC metastasis through Connectivity Map (Figure 8A). The structural formula and molecular formula of targeted drugs with the most potential value were obtained through PubChem22, including 5224221, calmidazolium, sulfasalazine, carbenoxolone, and tribenoside (Figures 8B–F).

### Validation of the Expression of mIR-DEGs in Clinical Tissue Samples

**FIGURE 9:**
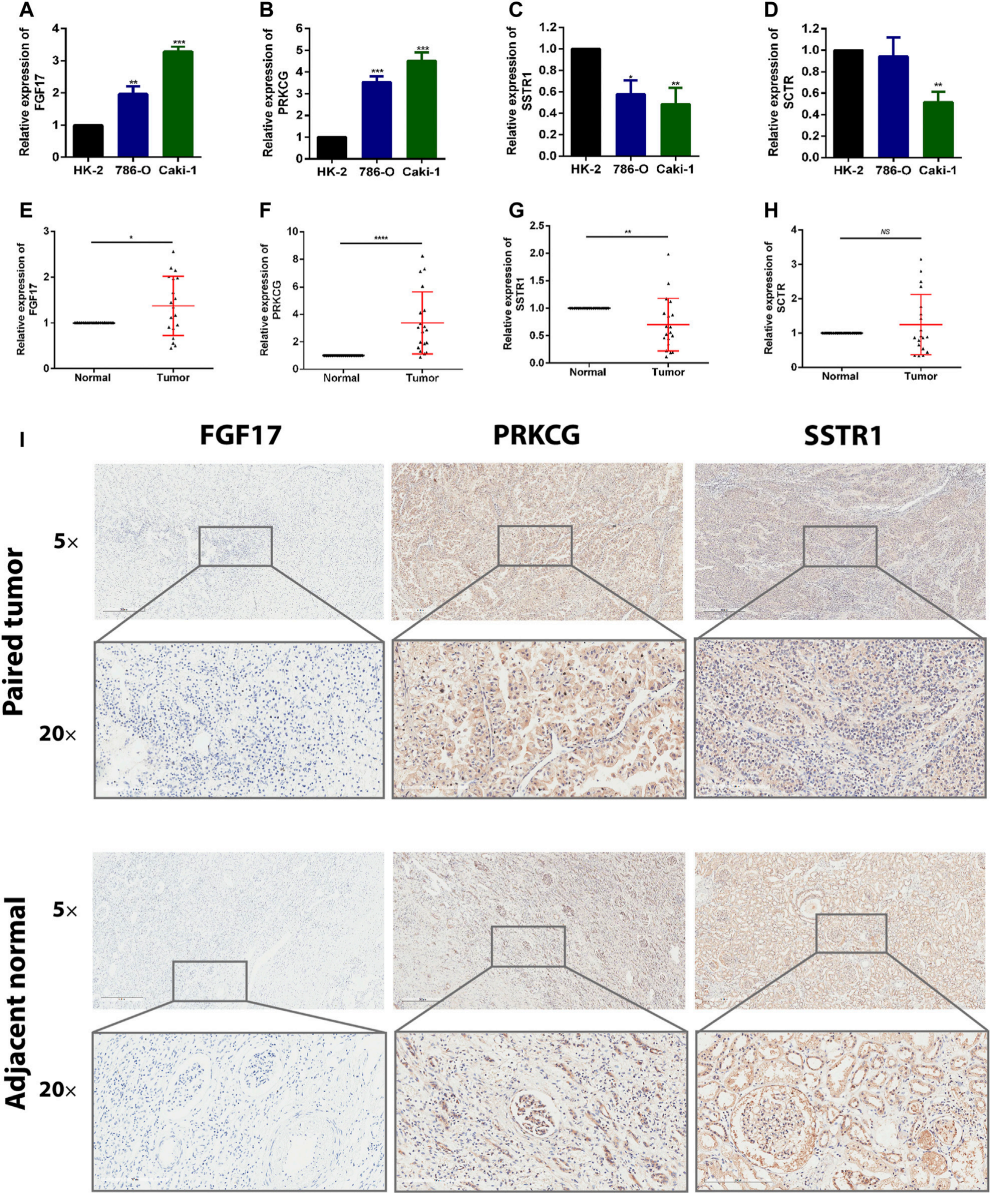
Expression of these hub genes in human KIRC specimens, adjacent normal tissues, and cell lines. **(A–D)** qRT-PCR analysis of FGF17 **(A)**, PRKCG **(B)**, SSTR1 **(C)**, and SCTR **(D)** in KIRC cell lines. GAPDH was used as a loading control. **(E–H)** qRT-PCR analysis of FGF17 **(E)**, PRKCG **(F)**, SSTR1 **(G)**, and SCTR **(H)** in paired KIRC tissues (*n* = 19). **(I)** Representative images of FGF17, PRKCG, and SSTR1 protein immunochemistry in KIRC tissues compared with adjacent normal kidney tissues. Magnification: ×50, ×200; *p < 0.05, **p < 0.01, ***p < 0.001.

To detect the expression of four genes (FGF17, PRKCG, SSTR1, and SCTR) in KIRC, we performed the qRT-PCR in KIRC cells and clinical tissue samples. We verified the expression levels of four genes in the normal kidney cell line (HK-2 cells) and two KIRC cell lines (786-O, caki-1). The results showed that the expression levels of FGF17 and PRKCG were significantly increased in KIRC cells compared with normal kidney cells, while SSTR1 and SCTR were down-regulated in KIRC cells (Figures 9A–D). FGF17, PRKCG, and SSTR1 were detected with the same results in tumor tissues and adjacent normal kidney tissues, while SCTR was not significantly different (Figures 9E–H). Then, we detected the protein expression of FGF17, PRKCG, and SSTR1 in the tissues by IHC. The IHC results showed that PRKCG was strongly expressed in the cytoplasm of KIRC tissues compared with adjacent normal kidney tissues. The expression of SSTR1, which was mainly expressed in the cytosol and cytoplasm, was significantly decreased. FGF17 positive expression was mainly distributed extracellularly, but FGF17 was negative in most tissues (Figure 9I).

## Discussion

Renal cell carcinoma was one of the most common urinary system tumors; about 25–30% of patients were metastatic at initial diagnosis, and 20–30% of patients had a tendency of recurrence and metastasis after local surgery, especially ccRCC (Jung et al., 2001). Many studies had shown that, in mRCC, the top three metastases were lung (45–75%), bone (15–34%), and liver (20%), whose five-year survival rates were 36–50%, 35%, and 18–43%, respectively (Staehler, 2011; Hatzaras et al., 2012). Given the rapid development of tumor immunology, a large number of previous studies had found that traditional immunotherapy, such as IFN-α and IL-2, could extend the OS to a certain extent, but their response duration was limited, and only a few patients could fully respond (Floros and Tarhini, 2015; Mao et al., 2021b). Currently, new immunotherapy drugs have been developed successively, such as cancer vaccine (Amin et al., 2015), adoptive cell therapy (Tang et al., 2013), and checkpoint inhibitors (Ghatalia et al., 2017). These drugs were reported to be capable of prolonging the response time of combination drugs and improving the OS significantly. Therefore, it was of great significance to elucidate the molecular mechanism of immune-related invasion and metastasis of RCC and to identify potential biomarkers for immunotherapy in RCC.

In this study, we firstly screened in GSE12606, GSE47352, and immune-related genes to analyze the co-expression of differential genes in primary and metastatic renal carcinoma. Secondly, FGF17, PRKCG, SSTR1, and SCTR were identified as metastatic immune-related independent risk factors by differential expression analysis, prognostic analysis, and univariate and multivariate Cox regression analysis. Then, the risk prognostic model was constructed based on lasso regression analysis, that is, Risk Score= (−0.1637) × SCTR + (−0.2632) × SSTR1 + (0.1711) PRKCG + (0.7824) FGF17. The predictive value of this model was favorable. There were significant correlations between the expression levels of four mIR-DEGs and clinicopathological factors, immune infiltration, and immune checkpoint. In addition, the calibration curves and nomogram showed an excellent prediction effect. Subsequently, through OCLR scores, it was further confirmed that the expressions of FGF17, PRKCG, SSTR1, and SCTR were different in KIRC, which might lead to tumor metastasis by promoting tumor dedifferentiation. Therefore, all of these results preliminary indicate that FGF17, PRKCG, SSTR1, and SCTR may impact the progression and metastasis in KIRC. Furthermore, their significant association with KIRC immune microenvironment and immune checkpoint–related genes also implied that mIR-DEGs may be potential targets and prognostic biomarkers for KIRC immunotherapy.

FGF17, as a member of the fibroblast growth factor (FGF) family, was located at 8p21.3 and played a significant role in the occurrence and progression of cancer (Tabarés-Seisdedos and Rubenstein, 2009). Studies had shown that the dual inhibition of FGF and CSF1 or VEGF signals was expected to enhance the antitumor effect by targeting immune escape and angiogenesis in the tumor microenvironment (Katoh, 2016). Protein kinase C gamma (PRKCG), as an isoenzyme of protein kinase Cs (PKCs) (Nishizuka, 1984), mediates IL-2 expression and tumor immune response (Chen et al., 1994). The 20th serine site could also be phosphorylated in p53 to activate apoptosis of colon cancer cells (Kawabata et al., 2012). Somatostatin receptor 1 (SSTR1) was a subtype of SSTR, belonging to the G-protein–coupled receptor (GPCR), which was involved in various signal transduction mechanisms in different parts of the human body (Nagarajan et al., 2020). Studies had found abnormal expression of SSTR in prostate cancer, colorectal cancer, breast cancer, and leiomyoma (Reubi et al., 1998), and high expression of SSTR1 could reduce the proliferation of acetaldehyde dehydrogenase (ALDH) positive cells, resulting in silenced and proliferation inhibition of colon cancer stem cells (Zou Y et al., 2019). Therefore, somatostatin analogs (SSAs) had been studied for immunotherapy of various cancers (Li et al., 2005). Secretin receptor (SCTR), also known as GPCR, was abnormally expressed in many cancers to affect the proliferation of tumor cells (Awasthi et al., 2012). Low expression of SCTR could stimulate tumor cell proliferation through the PI3K/AKT signaling pathway (Lee et al., 2012), and the combination of PI3K inhibitors and tumor chemoradiotherapy had been shown to inhibit tumor proliferation. In summary, we found significant differences in the expression of FGF17, PRKCG, SSTR1, and SCTR in cancer, which are correlated with immune response and adjuvant therapy. However, the specific functions and potential mechanisms of these four immune-related genes in KIRC metastasis remained unclear and needed further exploration. In addition, we performed qRT-PCR analysis on clinical specimens and found that the mRNA expression levels of FGF17, PRKCG, and SSTR1 were significantly different between kidney cancer tissues and normal tissues adjacent to the cancer. However, more *in vivo* and *in vitro* experiments are needed to confirm these findings.

Interestingly, the gene probes targeting FGF17, PRKCG, SSTR1, and SCTR predicted potential targeted agents for renal cancer metastasis and adjuvant immunotherapy, including 5224221, calmidazolium, and sulfasalazine. Current studies had found that calmidazolium, as calmodulin inhibitors, could not only affect the survival status of various immune cells (Hu et al., 2019) but also affect the inositol-1,4,5-triphosphate receptor/calcium/calmodulin pathway by mediating RACK1 and regulate the proliferation of preglomerular microvascular smooth muscle cells and mesangial cells, thus treating kidney diseases (Cheng et al., 2011). In addition, calmidazolium can induce apoptosis and down-regulate stem cell–related genes to inhibit the growth of embryonal carcinoma cells (Lee et al., 2016). Sulfasalazine, as sulfonamide antibiotic, had antibacterial, anti-inflammatory, and immuno-suppressive effects. Studies had shown that sulfasalazine could be involved in cancer cell death and T cell immunity by inhibiting the ferroptosis-related NF-κB signaling pathway and systemic Xc transporters (Dixon et al., 2012; Dixon et al., 2014). At present, sulfasalazine had been found to have significant effects on tumor cells in breast cancer (Yu et al., 2019), thyroid cancer (Zou L et al., 2019), kidney cancer (Sourbier et al., 2007), and bladder cancer (Ogihara et al., 2019). Although calmidazolium and sulfasalazine had been proven to affect the occurrence, metastasis, and apoptosis of various tumors, their specific mechanisms were still unclear, and there was no relevant study on the efficacy in KIRC, which was worth further exploration.

There are also some limitations in this study. First, the retrospective study determined that there is heterogeneity in the results, so further *in vivo* and *in vitro* experiments are needed to validate the findings of this study. Second, it is necessary that we need more basic and large clinical trials to validate these findings.

## Conclusion

In this study, we obtained hub mIR-DEGs with prognostic value through comprehensive bioinformatics analysis, including FGF17, PRKCG, SSTR1, and SCTR, which were significantly associated with methylation, ferroptosis, and immune checkpoint–related genes in KIRC. Preliminary validation found that PRKCG and SSTR1 were consistent with predictions. These indicators could be new targets and prognostic biomarkers for KIRC’s metastasis and immunotherapy. Furthermore, we had predicted the formula of targeted small molecule drugs based on hub mIR-DEGs. However, this prediction still needed lots of basic experimental demonstration.

## Data Availability

The datasets presented in this study can be found in online repositories. The names of the repository/repositories and accession number(s) can be found in the article/Supplementary Material.
