# Supplementary material for: Metastatic Immune-Related Genes for Affecting Prognosis and Immune Response in Renal Clear Cell Carcinoma
Source: Front Mol Biosci. 2022 Jan 28;8:794326. doi: 10.3389/fmolb.2021.794326 (PMC8832145; doi:10.3389/fmolb.2021.794326)
Supplement: Supplementary file 1 [file Datasheet1.DOCX]

**Table 1 | Basic information about GSE datasets.**

| Dataset | Number of samples | Array types | Tissue |
| --- | --- | --- | --- |
| ( Primary /Metastatic) | | | |
| GSE12606 | 3 primary and 3 metastatic samples | GPL570(HG‑U133_Plus_2) | clear cell renal cell carcinoma |
| GSE47352 | 5 primary and 4 metastatic samples | GPL570(HG‑U133_Plus_2) | clear cell renal cell carcinoma |

**Table 2 |** **basic information of TCGA-KIRC patients.**

| **Characteristic** | **Population** | | | |
| --- | --- | --- | --- | --- |
| n | 539 | | | |
| T stage | T1 | T2 | T3 | T4 |
| T1 | 278 | 71 | 179 | 11 |
| N stage | N0 | N1 |  |  |
|  | 241 | 16 |  |  |
| M stage | M0 | M1 |  |  |
|  | 428 | 78 |  |  |
| Gender | Female | Male |  |  |
|  | 186 | 353 |  |  |
| Pathologic stage | Stage I | Stage II | Stage III | Stage IV |
|  | 272 | 59 | 123 | 82 |
| Primary therapy outcome | PD | SD | PR | CR |
|  | 11 | 6 | 2 | 128 |
| Race | Asian | Black or African American | White |  |
|  | 8 | 57 | 467 |  |
| Age | <=60 | >60 |  |  |
|  | 269 | 270 |  |  |
| Histologic grade | G1 | G2 | G3 | G4 |
|  | 14 | 235 | 207 | 75 |
| Laterality, n (%) | Left | Right |  |  |
|  | 252 | 286 |  |  |

**Table 3 | Clinical characteristics of 19 KIRC patients.**

| Sample Number | Age | Gender | T | N | M | Fuhrman | Tumor size（cm） |
| --- | --- | --- | --- | --- | --- | --- | --- |
| 1 | 59 | Female | 1 | 0 | 0 | I | 3 |
| 2 | 74 | Male | 3 | 0 | 0 | II | 3.1 |
| 3 | 52 | Male | 1 | 0 | 0 | II | 6 |
| 4 | 78 | Female | 3 | 0 | 0 | III | 8.5 |
| 5 | 82 | Female | 3 | 0 | 0 | II | 4 |
| 6 | 54 | Male | 1 | 1 | 0 | I | 2.5 |
| 7 | 46 | Male | 3 | 0 | 1 | IV | 16 |
| 8 | 64 | Male | 1 | 0 | 0 | III | 3 |
| 9 | 23 | Male | 1 | 0 | 0 | II | 2 |
| 10 | 82 | Female | 1 | 0 | 0 | III | 3.3 |
| 11 | 77 | Male | 1 | 0 | 0 | II | 3.4 |
| 12 | 68 | Male | 1 | 0 | 0 | II | 0.8 |
| 13 | 43 | Male | 4 | 0 | 0 | II | 9.5 |
| 14 | 65 | Female | 3 | 1 | 0 | IV | 10 |
| 15 | 70 | Female | 2 | 0 | 0 | II | 9 |
| 16 | 58 | Male | 1 | 0 | 0 | II | 4.3 |
| 17 | 74 | Female | 1 | 0 | 0 | II | 2 |
| 18 | 40 | Male | 3 | 0 | 1 | III | 10.7 |
| 19 | 44 | Male | 1 | 0 | 0 | I | 1.8 |

**Table 4| The primer sequences used in the present study.**

| Gene | Forward primer (5′-3′) | Reverse primer (5′-3′) |
| --- | --- | --- |
| FGF17 | CTGCTGATTCTCTGCTGTCAA | GTAGAGTTGGTACTCGCGGAT |
| PRKCG | AGCCACAAGTTCACCGCTC | GGACACTCGAAGGTCACAAAT |
| SSTR1 | GCGCCATCCTGATCTCTTTCA | AACGTGGAGGTGACTAGGAAG |
| SCTR | CCCTTCCCCGACTATGTGAC | TGCAGTTTCGGAACAAGGAAC |
| GAPDH | ACCATCTTCCAGGAGCGAGAT | GGGCAGAGATGATGACCCTTT |

**Table 5** | **GO and KEGG enrichments**. Bioinformatics analysis of 14 IR-DEGs in KIRC.

| ONTOLOGY | ID | Description | GeneRatio | BgRatio | pvalue | p.adjust | qvalue |
| --- | --- | --- | --- | --- | --- | --- | --- |
| BP | GO:0048806 | genitalia development | 3/14 | 47/18670 | 5.34e-06 | 0.002 | 7.52e-04 |
| BP | GO:0010862 | positive regulation of pathway-restricted SMAD protein phosphorylation | 3/14 | 48/18670 | 5.69e-06 | 0.002 | 7.52e-04 |
| BP | GO:0048608 | reproductive structure development | 5/14 | 431/18670 | 1.08e-05 | 0.002 | 7.52e-04 |
| BP | GO:0061458 | reproductive system development | 5/14 | 434/18670 | 1.12e-05 | 0.002 | 7.52e-04 |
| BP | GO:0060393 | regulation of pathway-restricted SMAD protein phosphorylation | 3/14 | 62/18670 | 1.24e-05 | 0.002 | 7.52e-04 |
| MF | GO:0008528 | G protein-coupled peptide receptor activity | 4/14 | 146/17697 | 4.17e-06 | 1.54e-04 | 5.91e-05 |
| MF | GO:0001653 | peptide receptor activity | 4/14 | 152/17697 | 4.90e-06 | 1.54e-04 | 5.91e-05 |
| MF | GO:0008083 | growth factor activity | 4/14 | 163/17697 | 6.46e-06 | 1.54e-04 | 5.91e-05 |
| MF | GO:0005160 | transforming growth factor beta receptor binding | 3/14 | 51/17697 | 8.03e-06 | 1.54e-04 | 5.91e-05 |
| MF | GO:0070700 | BMP receptor binding | 2/14 | 11/17697 | 3.18e-05 | 4.90e-04 | 1.88e-04 |
| KEGG | hsa04014 | Ras signaling pathway | 4/13 | 232/8076 | 3.87e-04 | 0.022 | 0.018 |
| KEGG | hsa04350 | TGF-beta signaling pathway | 3/13 | 94/8076 | 4.01e-04 | 0.022 | 0.018 |
| KEGG | hsa04010 | MAPK signaling pathway | 4/13 | 294/8076 | 9.48e-04 | 0.026 | 0.021 |
| KEGG | hsa04060 | Cytokine-cytokine receptor interaction | 4/13 | 295/8076 | 9.60e-04 | 0.026 | 0.021 |
| KEGG | hsa04390 | Hippo signaling pathway | 3/13 | 157/8076 | 0.002 | 0.039 | 0.031 |

**Table 6 | Univariate and multivariate Cox regression analyses of 14 IR-DEGs for OS in the entire TCGA cohort.**

| Characteristics | Total(N) | Univariate analysis | |  | Multivariate analysis | |
| --- | --- | --- | --- | --- | --- | --- |
|  |  | Hazard ratio (95% CI) | P value |  | Hazard ratio (95% CI) | P value |
| PTPN11 | 539 |  |  |  |  |  |
| High | 269 | Reference |  |  |  |  |
| Low | 270 | 1.574 (1.159-2.137) | **0.004** |  | 1.041 (0.725-1.495) | 0.828 |
| CETP | 539 |  |  |  |  |  |
| High | 269 | Reference |  |  |  |  |
| Low | 270 | 1.558 (1.150-2.112) | **0.004** |  | 1.157 (0.808-1.656) | 0.427 |
| BMP5 | 539 |  |  |  |  |  |
| High | 269 | Reference |  |  |  |  |
| Low | 270 | 1.346 (0.995-1.820) | 0.054 |  | 1.032 (0.732-1.455) | 0.856 |
| ESR2 | 539 |  |  |  |  |  |
| High | 269 | Reference |  |  |  |  |
| Low | 270 | 0.802 (0.594-1.082) | 0.149 |  |  |  |
| SCTR | 539 |  |  |  |  |  |
| High | 269 | Reference |  |  |  |  |
| Low | 270 | 1.396 (1.032-1.889) | **0.030** |  | 1.842 (1.318-2.573) | **<0.001** |
| NR6A1 | 539 |  |  |  |  |  |
| High | 269 | Reference |  |  |  |  |
| Low | 270 | 1.078 (0.799-1.454) | 0.622 |  |  |  |
| FLT1 | 539 |  |  |  |  |  |
| High | 269 | Reference |  |  |  |  |
| Low | 270 | 1.859 (1.365-2.533) | **<0.001** |  | 1.201 (0.802-1.797) | 0.374 |
| BMP6 | 539 |  |  |  |  |  |
| High | 269 | Reference |  |  |  |  |
| Low | 270 | 1.670 (1.230-2.266) | **<0.001** |  | 1.145 (0.798-1.643) | 0.462 |
| SSTR1 | 539 |  |  |  |  |  |
| High | 269 | Reference |  |  |  |  |
| Low | 270 | 2.087 (1.527-2.853) | **<0.001** |  | 1.743 (1.235-2.460) | **0.002** |
| PRKCG | 539 |  |  |  |  |  |
| High | 269 | Reference |  |  |  |  |
| Low | 270 | 0.492 (0.361-0.671) | **<0.001** |  | 0.577 (0.410-0.812) | **0.002** |
| FGF17 | 539 |  |  |  |  |  |
| High | 269 | Reference |  |  |  |  |
| Low | 270 | 0.658 (0.486-0.890) | **0.007** |  | 0.595 (0.416-0.851) | **0.004** |
| TGFB2 | 539 |  |  |  |  |  |
| High | 269 | Reference |  |  |  |  |
| Low | 270 | 1.404 (1.034-1.905) | **0.030** |  | 1.101 (0.771-1.571) | 0.597 |
| CXCR3 | 539 |  |  |  |  |  |
| High | 269 | Reference |  |  |  |  |
| Low | 270 | 0.930 (0.690-1.253) | 0.632 |  |  |  |
